# Concentric energy transfer with quantum dots for multiplexed biosensing

**DOI:** 10.3402/nano.v4i0.22428

**Published:** 2013-09-03

**Authors:** W. Russ Algar, Igor L. Medintz

**Affiliations:** 1 Department of Chemistry, University of British Columbia, Vancouver, BC, Canada; 2 Center for Bio/Molecular Science and Engineering, U.S. Naval Research Laboratory, Washington, DC, USA

## Building smarter bioprobes using FRET relays

Colloidal semiconductor quantum dots (QDs) and their bioconjugates are of great interest for bioanalysis and bioimaging. These brightly luminescent nanoparticles can be utilised as probes to light-up biological structures and report on biochemical processes, potentially providing new insight into fundamental biology or enabling better diagnostic methods. Some of the advantages of QDs in these applications include their spectrally narrow, size-tunable emission; broad, strong light absorption; resistance to photobleaching; and facile multicolour imaging and analysis. Over the last decade, a multitude of QD bioprobes based on Förster resonance energy transfer (FRET) have been developed, where FRET is used to turn QD luminescence ‘on’ or ‘off’ in response to binding of biological targets or other bioprocesses of interest.

Algar and coworkers have recently developed a QD-based ‘concentric’ energy transfer relay with new and promising capabilities (1). Two peptide sequences, labelled at their distal termini with either a yellow emitting fluorescent dye, 1 (Alexa Fluor 555), or a deep red emitting fluorescent dye, 2 (Alexa Fluor 647), are self-assembled to a central CdSe/ZnS QD with green emission. As pictured, following optical excitation of the QD, three possible FRET pathways exist within this configuration: 1) from the QD to dye 1, 2) from the QD to dye 2, and 3) a relay from dye 1 to dye 2 after initial energy transfer from the QD. The ‘concentric’ nomenclature arises from the positioning of the two fluorescent dyes on the surface of an imaginary sphere, approximately 10 nm in diameter, surrounding a 5.6 nm diameter QD.

One application for the concentric FRET relay is multiplexed sensing of protease activity. Abnormal proteolysis is associated with diseases such as Alzheimer's, arthritis, and various types of cancers. The combination of the three emission signals from the QD, dye 1, and dye 2 can be uniquely correlated to the number of peptides labelled with dye 1 and dye 2 per QD. When the peptides are selected to be substrates for two different proteases, the hydrolytic activity of those enzymes can be quantitatively tracked, including the activation of an inactive pro-protease by an upstream protease. Cascades such as the latter are how many proteolytic signalling pathways operate in biological systems.

**Figure:**
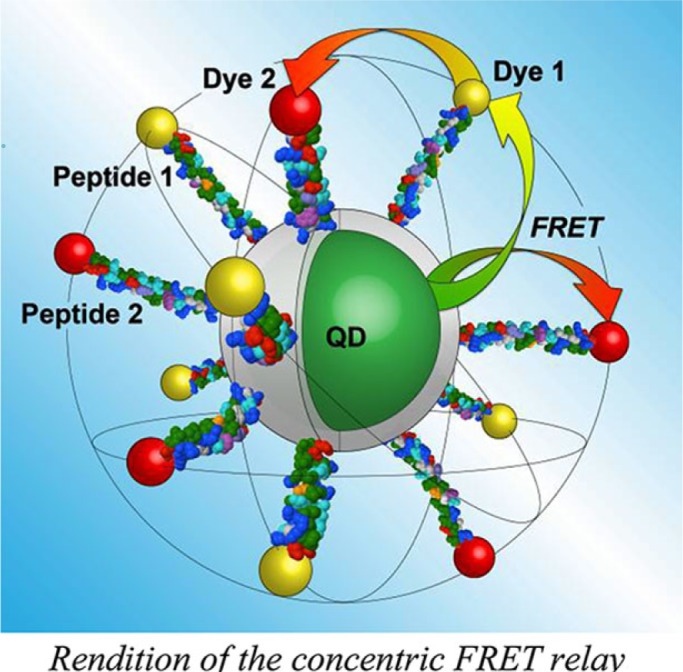


An exciting feature of the concentric FRET relay is that only one type of nanoparticle vector is needed to track the activity of two proteases, both in the ensemble and, in principle, at the level of single QDs. The configuration is uniquely enabled by the surface area of the central QD, which acts as a scaffold upon which multiple-dye-labelled peptides can be assembled, and also the concentricity, which permits varying degrees of energy transfer from the QD to both dyes, with or without the inter-dye relay. Such an arrangement would not be practically feasible with a fluorescent dye or fluorescent protein as the initial donor in the FRET relay. Importantly, the surface area of the QD will also be able to accommodate cell-penetrating peptides or other targeting ligands that are critical for prospective sensing applications in cells or tissues. Further development of the concentric QD-FRET relay is ultimately expected to provide opportunities for visualising cellular enzymatic processes with new levels of detail.
